# Supplementary material for: The Effect of Heat Treatment toward Glycerol-Based, Photocurable Polymeric Scaffold: Mechanical, Degradation and Biocompatibility
Source: Polymers (Basel). 2021 Jun 14;13(12):1960. doi: 10.3390/polym13121960 (PMC8232022; doi:10.3390/polym13121960)
Supplement: Supplementary file 1 [file polymers-13-01960-s001.zip › polymers-1252059-supplementary.pdf]

Supplementary information

The Effect of Heat Treatment toward Glycerol-Based, Photocurable Polymeric Scaffold: Mechanical, Degradation, and Biocompatibility

Wai-Sam Ao-Ieong [1], Shin-Tian Chien[1], Wei-Cheng Jiang[2], Shaw-Fang Yet[2], Jane Wang[1]

[1] *Department of Chemical Engineering, National Tsing Hua University, Hsinchu, Taiwan*

[2] *Institute of Cellular and System Medicine, National Health Research Institutes, Zhunan, Taiwan*

Number of Tables: 0

Number of Figures: 3

Number of pages: 4

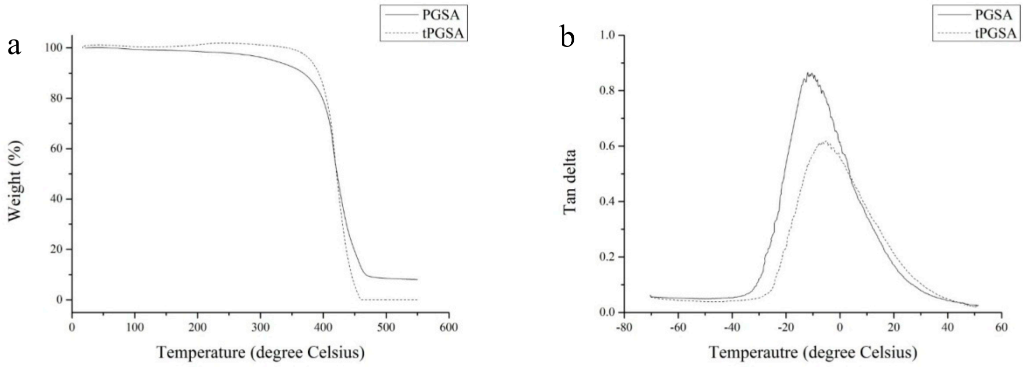

Figure S1 Thermal analysis of PGSA and tPGSA. The decomposition temperature and glass transition temperature were evaluated through (a) TGA analysis and (b) DMA analysis, respectively.

According to Figure S1a, the decomposition temperature of PGSA and tPGSA was 416.2 ˚C and 417.6 ˚C, respectively. This result suggested that the overall composition of the polymer networks was consistency between PGSA and tPGSA. In Figure S1a, the weight of PGSA was slightly decreased when the temperature was below 300 ˚C, suggesting that some unreacted chemicals and small chains were evaporated from the polymer network. In contrary to PGSA, no significantly change was observed on the weight of tPGSA below 300 ˚C, indicating that the thermally treated polymer network was stable before the temperature reached to the decomposition temperature. In Figure S1b, the peaks of tan delta for PGSA and tPGSA, which is also known as "glass transition temperature (Tg)", were -11.9 ˚C and -5.197 ˚C, respectively. The increase Tg of PGSA after thermal treatment was due to the formation of new crosslinks in the polymer network, while the similar effect of different crosslinking density on styrene-butadiene rubbers was also observed by Bandzierz et al. [1]. According to the above result, PGSA and tPGSA was stable under room temperature.

a

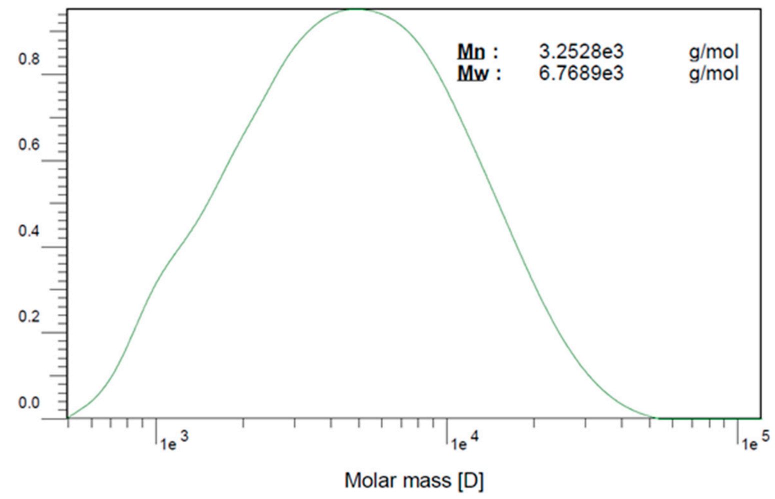

b

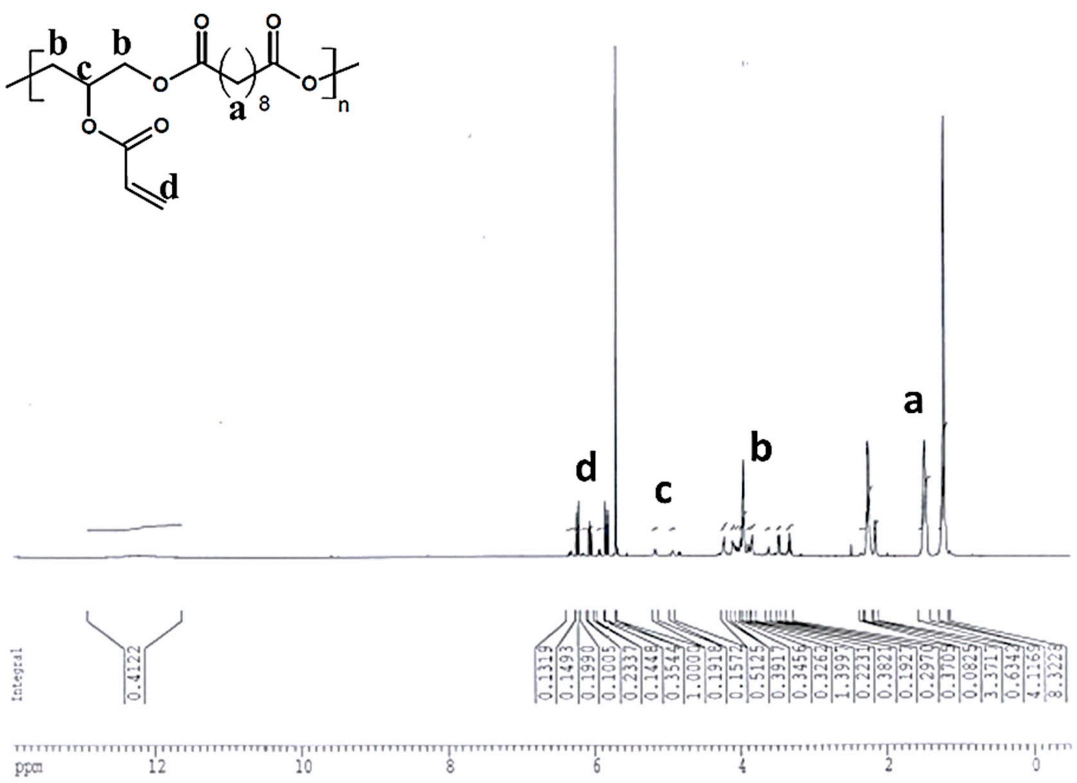

Figure S2 Characterization of PGSA pre-polymer. (a) The molar mass distribution of PGSA pre-polymer through GPC, and (b) ^{1}H-NMR spectrum of PGSA pre-polymer for the calculation of degree of acylation.

First, v_2 and V_1 were obtained for PGSA and tPGSA through the swelling ratio in water (Fig. 3b, Day 0). As the composition of bonding between PGSA and tPGSA polymer networks is largely unchanged, it is assumed that x_1 of PGSA is very similar to tPGSA. Therefore, through the Flory-Rhener equation (eq S1), the ratio between the number of network chain segments of tPGSA to PGSA was 1.42 (eq S2).

$$-[\ln(1-v_2)+v_2+x_1 v_2^2] = V_1 n(v_2^{\frac{1}{3}}-\frac{v_2}{2})\text{.........eq S1}$$

$$\frac{n_{tPGSA}}{n_{PGSA}} = 1.42\text{......eq S2}$$

where v_2 is the volume fraction of polymer in the swollen mass, V_1 is the molar volume of the solvent, n is the number of network chain segments bounded on both ends by crosslinks, x_1 is the Flory solvent-polymer interaction term.

As characterized through GPC, the average molecular weight of PGSA was nearly 3200 g/mol (Fig. S2a). According to the NMR spectrum (Fig. S2b), the degree of acrylation is evaluated to be 0.35 through the ratio of integral of the characteristic peaks between the acrylate groups and the sebacic acid. Therefore, it was calculated that a single PGSA linear chain contains around 12 repeating units, where 4 of them would be attached to an acrylate groups. Therefore, the average molecular mass between crosslinks (M_c) was assumed to be around 800 g/mol. Given that the density of PGSA and tPGSA films are 1.05 and 1.06, respectively. Through eq S3 from the Flory-Rhener eq, n_{PGSA} can be calculated as 5.95x10^{-4} mol/cm^3 and n_{tPGSA} as 8.33x10^{-4} mol/cm^3.

$$n = \frac{1}{vM_c}(1-\frac{2M_c}{\mathrm{M}})\text{.........eq S3}$$

where v is the specific volume of the polymer, M is the primary molecular mass.

Therefore, it was found that M_c of PGSA will be around 664.6 g/mol, indicating that the crosslinking density went up by about 40% in tPGSA network than the original PGSA, leading to the increase in the mechanical strength.

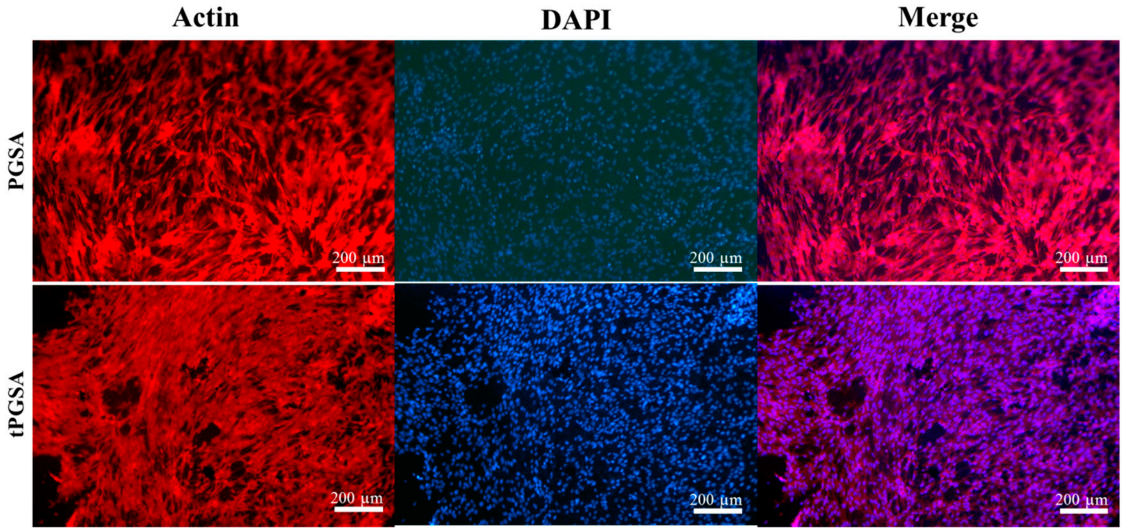

Figure S3 Fluorescent images of PGSA and tPGSA. Hig82 cells were seeding on PGSA and tPGSA films for 7 days before the staining.

Figure S3 is the Hig82 cell lines seeded on the PGSA and tPGSA films for 7 days. According to the figure, it is clear that cells on tPGSA obtained a higher proliferation than on PGSA. Moreover, cells were elongated and highly overlapped on both polymer surfaces.

Reference

1. Bandzierz, K., et al., *Influence of Network Structure on Glass Transition Temperature of Elastomers.* Materials (Basel, Switzerland), 2016. **9**(7): p. 607.
